# Hereditary Alpha-Tryptasemia and Mastocytosis: What We Know and What We Need To Learn

**DOI:** 10.1007/s11882-026-01262-9

**Published:** 2026-03-09

**Authors:** Tiago A. Rama, Theo Gulen

**Affiliations:** 1 Servico de Imunoalergologia, Unidade Local de Saúde de Matosinhos, Matosinhos, Portugal; 2 Serviço de Imunologia, Faculdade de Medicina da Universidade do Porto, Porto, Portugal; 3 Department of Respiratory Medicine and Allergy, Karolinska Mastocytosis Center, Karolinska University Hospital, Huddinge, Stockholm, Sweden; 4 Department of Medicine Solna, Division of Immunology and Allergy, Karolinska Institutet, Stockholm, Sweden; 5 Department of Medicine Huddinge, Clinical Lung and Allergy Research Unit, Karolinska Institutet, Stockholm, Sweden

**Keywords:** Hereditary alpha-tryptasemia, Tryptase, Anaphylaxis, systemic mastocytosis, MCAS

## Abstract

**Purpose of Review:**

This review aims to elucidate the evolving relationship between hereditary alpha-tryptasemia (HαT) and mastocytosis, emphasizing recent advances in understanding shared pathophysiological mechanisms, diagnostic challenges, and clinical implications.

**Recent Findings:**

HαT is a common genetic trait caused by increased copy numbers of the *TPSAB1* gene encoding alpha-tryptase. It accounts for most elevated serum baseline tryptase (SBT) levels (> 8 ng/mL) seen in clinical allergy practice. Diagnosis is established through tryptase genotyping—often referred to as “tryptase copy number variation (CNV) testing”.

Over the past decade, studies have reported a high prevalence of HαT among individuals with mastocytosis, prompting routine screening. However, the nature of this association remains controversial. While some studies suggest a potential link—most notably an increased risk of severe anaphylaxis—others have failed to show consistent correlations in pathophysiology or clinical outcomes. The evidence base is still evolving, and inconsistencies across studies underscore the need for cautious interpretation.

**Summary:**

HαT complicates the clinical assessment of mastocytosis by influencing baseline tryptase levels and potentially modulating symptom severity, though its clinical impact remains under investigation. Further research is needed to clarify the extent and nature of HαT’s contribution to mastocytosis and related mast cell disorders.

## Introduction

Mast cells (MCs) are tissue-resident immune cells derived from hematopoietic stem cells, strategically located near blood vessels, nerves, and mucosal surfaces [1, 2]. They serve as sentinels of the immune system, rapidly responding to environmental threats such as pathogens, toxins, and allergens [3, 4]. Upon activation—typically via IgE-mediated pathways—they release a cascade of bioactive mediators including histamine, cytokines, and proteases, which orchestrate inflammation, vascular permeability, and smooth muscle contraction [5, 6].

Among these mediators, tryptase is the most abundant mast cell-specific protease, stored in granules and released during degranulation. Tryptase exists as both α- and β-isoforms; α-tryptase is constitutively secreted and enzymatically inactive, whereas β-tryptase is stored in granules and released during mast-cell activation. It serves both as an effector molecule—contributing to tissue repair, vascular permeability, chemotaxis of neutrophils and eosinophils, thrombolysis, and amplification of allergic responses—and as a clinically valuable biomarker [7]. Serum tryptase is widely used to assess mast cell activation, particularly in the diagnosis of anaphylaxis and mast cell-related disorders. An acute rise in tryptase levels of at least 20% + 2 ng/mL over baseline [8] or; 68% over baseline during a symptomatic episode [9] is consistent with the diagnosis of a mast cell mediated systemic reaction (i.e., anaphylaxis) and is also used as one of the criteria for diagnosing mast cell activation syndrome (MCAS) [10]. Acute serum tryptase levels must be drawn no sooner than 30 min and no later than 4 h after symptom onset, ideally within the first 2 h of the event, otherwise transient elevations may be missed [11]. Heterogeneity has also been shown to exist within the mast cell compartment wherein certain mast cells contain more tryptase than others [12].

Elevated serum baseline tryptase (SBT) levels may indicate underlying conditions such as hereditary alpha-tryptasemia (HαT) or systemic mastocytosis (SM) [13]. HαT and SM are two distinct but increasingly recognized entities with relevance to mast cell biology. HαT is a genetic trait caused by increased copy number of the *TPSAB1* gene, which encodes either alpha- or beta-tryptase and leads to elevated SBT levels, often in the absence of overt disease [14]. SM, in contrast, is a clonal mast cell disorder involving dysregulated proliferation and activation, frequently associated with activating mutations in the *KIT* gene [15, 16]. It presents with a broad spectrum of clinical manifestations, ranging from indolent disease to aggressive systemic involvement [15, 16].

Recent studies suggest that HαT may be more prevalent among patients with mastocytosis than in the general population, raising questions about potential genetic and pathophysiological interactions [17]. Herein, we explore the clinical and mechanistic associations between HαT and mastocytosis, highlighting the clinical and diagnostic complexities posed by their co-occurrence. Understanding the biology of mast cells and the role of tryptase is essential for unravelling the complex interplay between these two conditions.

## Hereditary alpha-tryptasemia

HαT is inherited in an autosomal dominant fashion and affects up to 7.5% of individuals, particularly those of European descent [14, 18, 19]. It is caused by increased copy numbers of the alpha-tryptase–encoding gene *TPSAB1*, resulting in elevated SBT levels, typically above 8 ng/mL [20]. Unlike mastocytosis, HαT is not a clonal disorder and is considered a genetic trait rather than a disease. Referring to individuals with HαT as “patients” can be misleading and may pathologize what is, in many cases, a benign genetic variation.

HαT is associated with variations in tryptase gene copy number located on chromosome 16p13.3. This region contains four primary tryptase genes: *TPSB2* (encoding β-tryptase), *TPSAB1* (encoding either α- or β-tryptase), *TPSG1* (γ-tryptase), and *TPSD1* (δ-tryptase) [21, 22]. Among these, only α- and β-tryptases are secreted in clinically relevant quantities and are detectable in serum tryptase measurements [22]. Both α- and β-tryptase are initially synthesized as monomeric protryptase precursors, which may be constitutively secreted or processed into mature, enzymatically active tetramers [7]. The ELISA-based immunoassays used to analyse serum tryptase levels can detect the protryptase monomeric precursors and mature α- and β-tryptase forms [23].

**Fig. 1:**
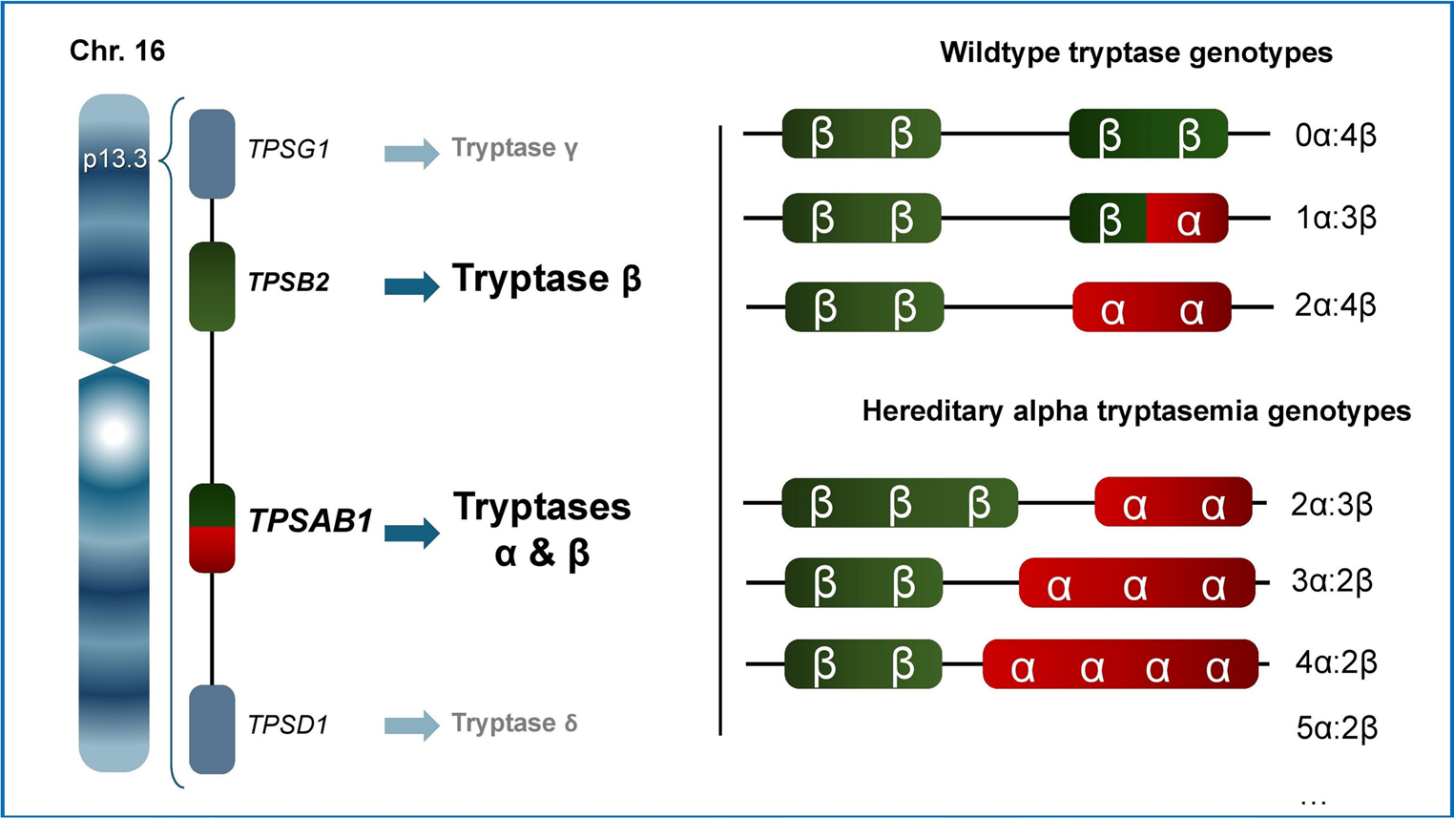
Inheritance patterns of HαT on chromosome 16p13.3 vary depending on parental haplotypes. HαT arises from an increased number of TPSAB1 copies encoding alpha tryptase on a single allele. Each individual inherits one haplotype from each parent, with variable numbers of α- and β-encoding TPSAB1 alleles. Wildtype genotypes include 0–2 α-tryptase alleles, whereas HαT is defined by extra copies of TPSAB1. Typical HαT variants involve duplications or triplications of alpha tryptase

Tryptases encoded by *TPSB2* (β) and *TPSAB1* (α or β) are inherited as a haplotype from each parent, with one allele from each gene passed on to the offspring [21]. In HαT, there is an excess of *TPSAB1* copy numbers encoding α-tryptase on one allele (Fig. 1). Diagnosis of HαT is based on two criteria: (1) elevated basal serum tryptase (> 8 ng/mL, often > 11 ng/mL), and (2) confirmed increased *TPSAB1* copy number via genetic testing.

**Fig. 2:**
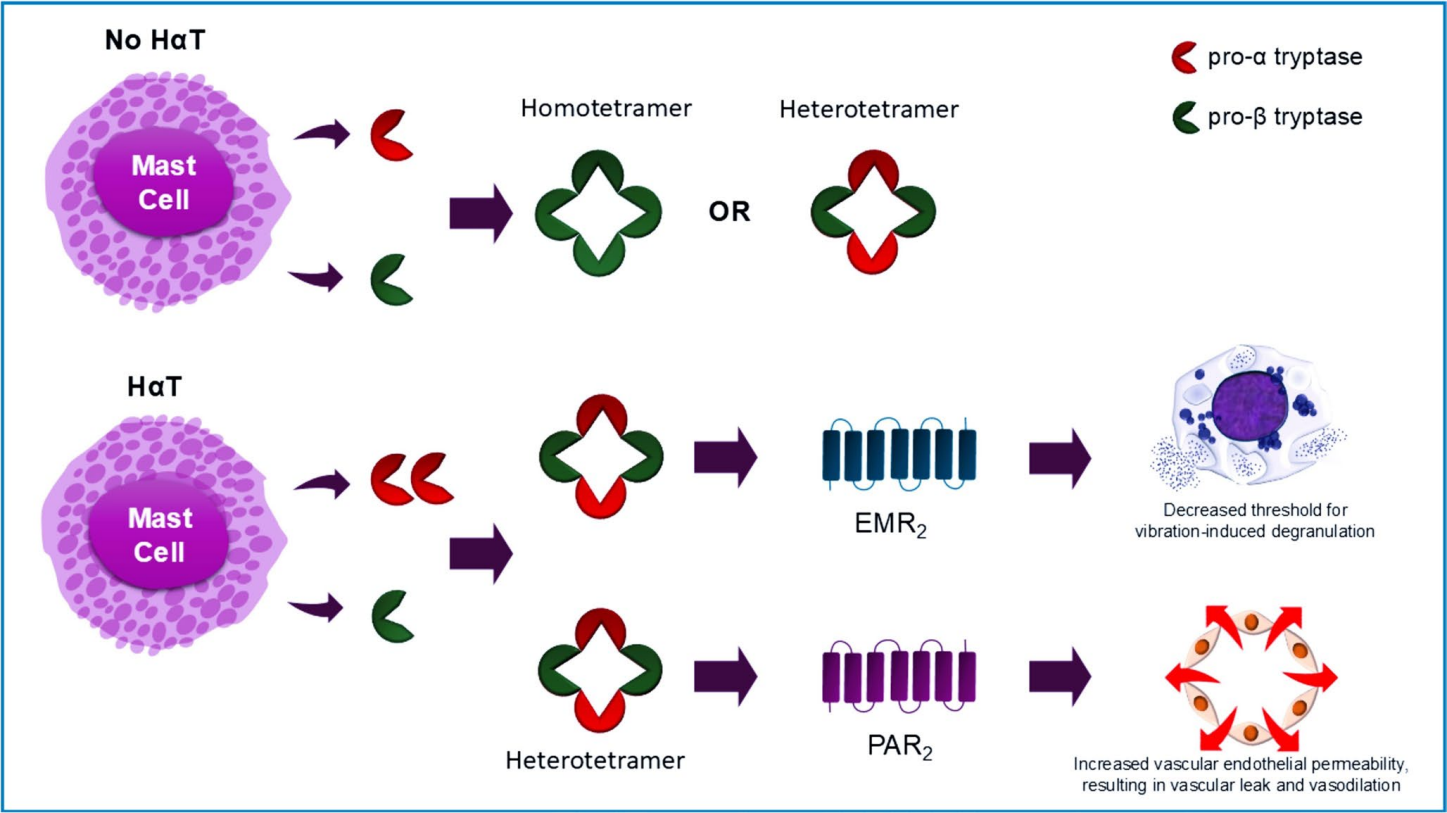
Schematic illustration of α/β-tryptase heterotetramers and their post-degranulation activity on EMR2 and PAR2 receptors. This figure depicts the structural arrangement of unique heterotetrameric complexes formed by α- and β-tryptase subunits following mast cell degranulation. Upon release, these tryptase tetramers interact with cell surface receptors, including EMR2 (EGF-like module-containing mucin-like hormone receptor-like 2) and PAR2 (protease-activated receptor 2), initiating downstream signalling pathways

Mature tryptases are tetrameric enzymes composed of either α-tryptase or β-tryptase subunits—or both, in the case of heterotetramers. Historically, enzymatically active tryptases were believed to exist only as β-tryptase homotetramers. Early cloning studies revealed that α-tryptase homotetramers lack enzymatic activity but are more stable and do not require heparin to maintain their quaternary toroidal structure [24]. In 2019, it was demonstrated that heterotetrameric tryptases containing both α- and β-subunits form naturally in humans, independent of HαT [25]. It has been postulated that α-tryptase–containing heterotetramers are more stable than β-tryptase homotetramers. These heterotetramers exhibit distinct biological activities, including cleavage and activation of EGF-like module-containing mucin-like hormone receptor-like 2 (EMR2), which may enhance mast cell activation in response to vibration, and activation of protease-activated receptor 2 (PAR2), which promotes vascular leak [25] (Fig. 2).

Although heterotetramer formation is not exclusive to HαT, individuals with increased α-tryptase gene copy number—characteristic of HαT—tend to produce more α/β-tryptase heterotetramers [25]. This elevated formation is associated with increased biological effects. In vitro, heterotetramers uniquely cleave EMR2, lowering the threshold for vibration-induced mast cell degranulation—an effect not observed with β-tryptase homotetramers [26]. MCs pretreated with heterotetramers degranulate in response to vibration, whereas those treated with homotetramers do not [25].

In addition to their role in mast cell activation, α/β-tryptase heterotetramers have been shown to increase vascular permeability via PAR2 activation [25, 27]. PAR2 is a G-protein-coupled receptor expressed on epithelial cells, smooth muscle cells, and neurons, and is implicated in regulating vascular basement membrane permeability [28, 29]. Exposure of human umbilical vein endothelial cells (HUVECs) to heterotetramers results in acute leak through a PAR2-dependent mechanism [22].

Taken together, these findings suggest that increased heterotetramer formation in HαT may contribute to symptom expression through both mast cell sensitization and vascular effects. Clinically, this raises the possibility that individuals with HαT—or more broadly, those with elevated α-tryptase levels—may be predisposed to hypotensive episodes and more severe anaphylactic reactions, although in vivo confirmation remains necessary [27].

The clinical relevance of HαT varies considerably. While many carriers remain asymptomatic, some individuals may present with symptoms such as flushing, pruritus, gastrointestinal discomfort, or recurrent hypersensitivity reactions—including urticaria, angioedema, or anaphylaxis [30, 31]. Notably, HαT has been linked to venom-induced and idiopathic anaphylaxis, with case reports and small studies showing elevated tryptase levels in the absence of mastocytosis [27]. While the association is biologically plausible, the current evidence remains limited and inconclusive.

In addition, connective tissue abnormalities, such as joint hypermobility, and autonomic dysfunction symptoms, including postural orthostatic tachycardia syndrome (POTS), have also been reported in certain cases; however, more recent studies have not consistently supported these specific associations [19, 32].

Notably, a gene-dose relationship has often been observed between TPSAB1 copy number and symptom severity in HαT, suggesting that individuals with more copies of the α-tryptase–encoding gene tend to have higher basal serum tryptase levels and may experience more pronounced clinical symptoms [33]. However, some studies have reported no consistent correlation between gene copy number and the frequency or intensity of clinical manifestations [34].

Taken together, studies reporting a broader range of symptom associations often rely on high-risk or referral populations, which may overrepresent such findings due to selection bias. Moreover, the presence of these symptoms does not imply that HαT is the cause.

## Mastocytosis

Mastocytosis refers to a complex heterogeneous multisystem disorder characterized by a pathologic activation and accumulation of clonally aberrant MCs in one or more organs, including the skin, bone marrow, liver, spleen, lymph nodes, and gastrointestinal tract [13, 15, 16]. The existing evidence suggests that it is a rare condition, and in recent studies, its prevalence is estimated to be 1 in 10,000 persons [35, 36]. The disease is broadly classified into cutaneous mastocytosis (CM), which is limited to the skin, and systemic mastocytosis (SM), where MCs infiltrate extracutaneous organs [13, 15, 16].

CM is the predominant form in children, typically presenting within the first year of life. The most common skin manifestation is maculopapular lesions, known as urticaria pigmentosa (UP) [13, 15, 16]. Pediatric CM is usually confined to the skin and resolves by adolescence. In contrast, adult-onset mastocytosis tends to be persistent and may or may not involve the skin. SM is diagnosed based on World Health Organization (WHO) criteria, which include one major criterion—multifocal dense MC aggregates in bone marrow or other extracutaneous tissues—and four minor criteria, such as atypical MC morphology, aberrant expression of CD2/CD25, detection of KIT D816V mutation, and elevated serum tryptase levels. A diagnosis requires either the major criterion plus one minor, or at least three minor criteria [15, 16] (Table 1). Rarely, adults may present with CM without systemic involvement. Indolent systemic mastocytosis (ISM) and bone marrow mastocytosis (BMM) represent the most prevalent subtypes of systemic mastocytosis (SM) in adults and are typically associated with normal life expectancy [35, 36]. In contrast, advanced SM variants—including aggressive SM (ASM), SM with an associated hematologic neoplasm (SM-AHN), and mast cell leukemia (MCL)—are rare and linked to poor prognosis due to a high burden of clonal mast cells [16, 36]. Notably, decreased survival in advanced SM is often driven by the associated hematologic neoplasm in SM‑AHN rather than by mast‑cell burden alone.

**Table 1:** Diagnostic criteria of systemic mastocytosis (SM), monoclonal mast cell activation syndrome (MMAS), mast cell activation syndrome (MCAS) and hereditary alpha-tryptasemia (HαT). (Adapted from references: 10, 13, 16)

SM	Diagnosis is confirmed if patient expresses one major criterion and one minor criterion or expresses three minor criteria in extracutaneous organ biopsy specimens
Major criterion	Multifocal aggregates of MCs (≥ 15 MCs per cluster) in biopsy sections
Minor criteria	1. In MC infiltrates in extracutaneous biopsy sections, > 25% of the MCs (CD117+) are spindle-shaped or have atypical morphology 2. Presence of an activating KIT mutation at codon 816, generally D816V, in bone marrow, blood, or other extracutaneous organ(s) 3. Detection of aberrant MC clones expressing CD117 with CD25 and/or CD2 and/or CD30 in bone marrow or blood or another extracutaneous organ(s) 4. *Baseline serum tryptase persistently exceeds ≥ 20 ng/mL
MMAS	Diagnosis requires presence of one or two minor criteria of SM
	1. Presence of an activating KIT mutation D816V, in bone marrow, blood or other extracutaneous organ(s) AND/OR 2. Detection of aberrant MC clones expressing CD117 with CD25 in bone marrow or blood or another extracutaneous organ(s)
MCAS	Diagnosis requires presence of one or twominor criteria of SM
	1. Severe, episodic symptoms that are attributable to MC activation with concurrent involvement of at least two organs, generally consistent with the diagnosis of recurrent anaphylaxis. 2. An event-related transient increase in serum tryptase above the individual’s SBT according to formula (≥ 1.2xSBT + 2 ng/mL) 3. Response to drugs directed against MC activation or effects of MC mediators to reduce/suppress symptoms
HαT	Diagnosis of HαT is based on two criteria
	1. Elevated serum baseline tryptase (≥ 8 ng/mL) 2. Confirmed increased *TPSAB1* copy number via genetic testing

HαT, hereditary alpha-tryptasemia; MCAS, mast cell activation syndromes; MMAS, monoclonal mast cell activation syndrome; SBT, serum baseline tryptase; SM, systemic mastocytosis

*In individuals with HαT, SBT should be adjusted for *TPSAB1* copy number before being considered a minor criterion [16, 37]

Beyond the WHO-defined SM variants, Monoclonal Mast Cell Activation Syndrome (MMAS) is a clonal disorder characterized by the presence of abnormal mast cells (e.g., expressing CD25 or carrying the KIT D816V mutation), without meeting full WHO criteria for SM [38–40]. In recent years, there has been a tendency to use the terms Monoclonal Mast Cell Activation Syndrome (MMAS) and Mast Cell Activation Syndrome (MCAS) interchangeably; however, not all patients with MMAS fulfil the consensus diagnostic criteria for MCAS [13] (Table 1). MMAS may present with clonal mast cells and elevated basal tryptase, yet lack sufficient clinical or biochemical evidence to meet the diagnostic thresholds established for MCAS. Consequently, alternative terminology for monoclonal mast cell activation syndrome (MMAS) has been sought, with ‘monoclonal mast cells with activation symptoms’ (MMAS) and ‘monoclonal mast cells with clinical significance’ (MMCS) emerging as options that better reflect the clonal nature of the condition without implying fulfilment of mast cell activation syndrome (MCAS) criteria [41, 42]. This distinction has been increasingly discussed in the literature, though formal consensus remains pending.

The clinical symptomatology of mastocytosis varies widely depending on the extent and the degree of mediator release. Common symptoms include flushing, pruritus, urticaria pigmentosa, abdominal pain, diarrhoea, nausea, and hypotension. Moreover, neurological symptoms such as headache, fatigue, and cognitive disturbances are also reported. Bone pain and osteoporosis may result from skeletal involvement [15, 43].

Anaphylaxis in mastocytosis is considerably more prevalent than in the general population, reaching up to 50% [13, 44, 45]. In specific subgroups of clonal mast cell disorders, such as MMAS, anaphylaxis is a hallmark clinical feature, occurring in 78% of patients [42]. In SM, prevalence is likewise elevated, particularly among individuals without skin lesions—most notably those with BMM, where rates may approach 90%—compared to approximately 25% in SM patients presenting with mastocytosis skin lesions [46]. Classically recognized risk factors for anaphylaxis may include atopy, high total IgE levels and low sBT levels in adults [47], while, in children, higher SBT [48, 49], extensive skin involvement [48], high lesional MC density and lesional blistering [50] may associate with anaphylaxis.

Anaphylaxis in adults with SM can be triggered by various factors including heat, cold, exercise, emotional stress, drugs, and Hymenoptera venom, or may occur spontaneously [44, 51, 52]. Among these, Hymenoptera stings are the most frequent elicitor, with a 28% prevalence of venom-induced anaphylaxis (VIA) in SM patients—significantly higher than in the general population [47]. VIA may even be the initial symptom leading to the SM diagnosis, as shown in a study where 10% of patients with Hymenoptera-induced anaphylaxis had elevated SBT) and were later diagnosed with SM or MMAS [53].

Food-induced anaphylaxis (FIA) is less common, with a prevalence at least ten times lower than VIA [54]. Most FIA cases are patient-reported and lack confirmatory testing. Additionally, while drug-induced anaphylaxis (DIA) has been considered a theoretical concern in mastocytosis, its prevalence appears somewhat higher than in the general population, yet remains low overall (6.3%) [55], and is much less frequent than VIA in mastocytosis. While certain drugs—such as NSAIDs, radiocontrast media, anaesthetics, and antibiotics—have been implicated in mast cell activation, systematic data do not support a high incidence of DIA in mastocytosis patients [55]. Moreover, recent cohort studies have reported NSAID-related anaphylaxis in only 3–9% of mastocytosis patients [56–58], and antibiotic-induced reactions in less than 1%, suggesting that routine drug avoidance may be overly cautious [59]. Conversely, idiopathic anaphylaxis (IA) is commonly seen in SM and may precede diagnosis [39, 44]. Its overlap with clonal mast cell disorders highlights the need for thorough evaluation to rule out underlying mastocytosis or MMAS.

A notable characteristic of anaphylaxis in mastocytosis is its atypical symptom profile. Patients frequently exhibit pronounced cardiovascular manifestations, particularly hypotensive syncope, while cutaneous and respiratory symptoms are uncommon. European data reveal that syncope occurs in the majority of SM-related anaphylaxis episodes—over 70%—regardless of the provoking agent [44, 46].

## Association Between HαT and Mastocytosis

### Prevalence and Diagnostic Implications

Emerging evidence indicates that HαT is significantly overrepresented in patients with systemic mastocytosis. Studies have shown that up to 15–20% of mastocytosis patients may carry extra copies of *TPSAB1*, compared to ~ 5–7% in the general population [17, 19, 26, 60–62]. Notably, one potential explanation for this higher prevalence may be that elevated SBT levels in individuals with HαT lead to bone marrow biopsy, particularly among those presenting with mast cell activation symptoms, thereby increasing the likelihood of diagnosing mastocytosis and contributing to the higher observed frequency of HαT within mastocytosis cohorts.

Beyond its potential impact on observed prevalence patterns, HαT also introduces important diagnostic challenges. Since elevated SBT levels are often the initial laboratory indicator raising suspicion of mastocytosis—particularly SM and MMAS, the presence of HαT can complicate the diagnostic interpretation. Individuals with HαT may exhibit chronically elevated SBT levels that mimic or obscure the biochemical signature of clonal mast cell disease, potentially delaying accurate diagnosis. In addition to affecting baseline levels, HαT can also interfere with the interpretation of tryptase dynamics during acute systemic mast cell activation events—i.e., anaphylaxis—where the relative rise may be less pronounced or difficult to distinguish from baseline variability. Although bone marrow biopsy remains the diagnostic gold standard for SM, elevated tryptase levels attributed to HαT may lead to deferral of this procedure, further postponing definitive diagnosis.

Accordingly, genetic testing for *TPSAB1* copy number variation may be considered in patients with unexplained elevated SBT, particularly when clinical features suggest mast cell activation but the likelihood of clonal mast cell disease is low. A positive result may help explain the biochemical findings and reduce the need for invasive diagnostic procedures. For instance, this approach may be relevant in patients with chronic spontaneous urticaria, where elevated tryptase levels may be present without other signs of clonal mast cell disease.

Together, these challenges underscore the need for integrated diagnostic algorithms that consider both genetic and clonal contributions to mast cell pathology.

### Clinical Manifestations and Modifier Role

The overlapping clinical features observed in a subset of individuals with HαT—such as flushing, gastrointestinal dysmotility, and cardiovascular symptoms—can complicate the differential diagnosis and obscure the underlying pathology, particularly when evaluating for mast cell activation or clonal mast cell disorders. Building on these clinical observations, recent studies have explored the role of HαT as a potential risk factor and modifier of mast cell activation symptoms in patients with SM.

In a multicenter cohort notably enriched with patients diagnosed with BMM from reference centers for Mast Cell Disorders in Italy, Slovenia, and the United States, Lyons et al. [27] demonstrated that individuals with both SM and HαT were 9.5 times more likely to experience VIA compared to those with wild-type tryptase genotypes. Another study from an Austrian Reference Center [17], involving a more diverse cohort including patients with CM and/or SM, found that mastocytosis patients with HαT were overall more symptomatic—particularly with regard to cardiovascular manifestations—and more prone to anaphylaxis, in particular VIA. Polivka et al. [60] further support the role of HαT as a clinical modifier in clonal mast cell disorders, highlighting its association with increased symptom severity and diagnostic challenges.

Additionally, an Italian multicenter study [61] investigating patients with MMAS or mastocytosis reported that BMM patients were disproportionately represented among those with HαT, comprising nearly half of the individuals with coexisting clonal mast cell disease and HαT. Notably, patients with HαT were also found to be more symptomatic, particularly with cardiovascular manifestations, and more prone to Hymenoptera venom allergy and anaphylaxis, suggesting that HαT may act as a clinical modifier in this subgroup.

### Conflicting Evidence and Phenotypic Variability

However, while several studies suggest that HαT may amplify clinical manifestations in clonal mast cell disorders, the evidence is not uniform across cohorts. For instance, Korosec et al. [63] reported that patients with both HαT and SM were more likely to exhibit a lower bone marrow mast cell burden and were less prone to show aggregates or spindle-shaped mast cells—features typically associated with a robust clonal phenotype—suggesting a potentially attenuated histopathologic presentation. This contrasts with other studies that link HαT to more severe clinical manifestations, such as increased anaphylaxis risk or heightened symptom burden. In the Korosec cohort, despite the presence of HαT, the clonal disease appeared histologically milder, raising questions about whether HαT consistently amplifies mast cell pathology or instead modulates it in a more variable, context-dependent manner. Their multicenter study across Central European Hymenoptera venom allergy (HVA) referral centers, which found approximately 8% of patients had HαT, 11% showed mast cell clonality, and 7% had neither, indicating a complex interplay between genetic predisposition and clonal disease expression.

Further complicating the picture, González-de-Olano et al. [62], studying a large Spanish cohort, reported that ~ 50% of patients with both HαT and clonal mast cell disease had BMM, and although HαT-positive clonal patients were more likely to present with anaphylaxis, the rate of subsequent anaphylactic events was similar between those with and without HαT. Importantly, this cohort did not show an increased prevalence of HαT among anaphylaxis patients, challenging the notion of HαT as a consistent enhancer of clinical severity.

### Methodological Considerations

Both studies [62, 63] used KIT D816V testing via allele-specific qPCR (ASOqPCR) in peripheral blood, a method with a false-negative rate of up to 50% [64, 65], which may have led to underestimation of clonality. Additionally, patients were referred irrespective of SBT levels, reducing referral bias and strengthening the generalizability of findings.

In the same line, a retrospective analysis by Chollet et al. [19], conducted at a U.S. academic allergy center as part of the PIONEER study, offers important methodological clarity. Within a rigorously phenotyped cohort of patients with ISM, the study found no significant association between HαT and anaphylaxis: rates were nearly identical between HαT-positive and HαT-negative individuals (18% vs. 17%). This finding suggests that when diagnostic criteria are standardized and cohort composition is tightly controlled, HαT may not independently predict anaphylaxis risk—highlighting the importance of methodological precision in evaluating genotype-phenotype relationships.

Additionally, Navarro-Navarro et al. [66] underscored the critical role of diagnostic sensitivity in detecting clonal mast cell disease. In their study of patients referred to Spain’s largest Reference Center—initially diagnosed using highly sensitive flow cytometry and ASO-qPCR—the proportion of HαT among BMM/MMAS patients increased when diagnosis was refined using ultra-sensitive techniques for KIT D816V detection in unfractioned bone marrow and blood. Interestingly, across multiple studies—Austrian [17], French [60], Italian [61], and Spanish [62] —HαT was consistently less frequent in patients with advanced SM compared to those with low bone marrow MC burden forms, and the proportion of HαT + and HαT– individuals was similar among those with advanced SM, suggesting a distinct genotype–phenotype relationship in low-burden clonal mast cell disorders.

Taken together, these methodological insights underscore the importance of diagnostic precision and cohort composition when evaluating the clinical impact of HαT in mast cell disorders. Table 2 provides a structured overview of how methodology, cohort composition, and diagnostic sensitivity shape the interpretation of HαT’s role — whether as a clinical amplifier, a context-dependent modifier, or a neutral factor.

**Table 2:** Summary of Studies Evaluating the Association Between Hereditary Alpha-Tryptasemia (HαT) and Anaphylaxis in Systemic Mastocytosis (SM)

Study	Cohort Description	Diagnostic Methods	Key Findings	Interpretation
Lyons et al. [27]	Multicenter cohort enriched with BMM patients (US, Italy, Slovenia)	Bone marrow KIT D816V testing; TPSAB1 genotyping	HαT associated with 9.5-fold increased risk of VIA in SM	Supportive
Austrian study [17]	Diverse cohort including CM and/or SM patients	Bone marrow KIT D816V testing; TPSAB1 genotyping	HαT linked to more symptomatic disease, especially cardiovascular symptoms and anaphylaxis	Supportive
Polivka et al. [60]	French cohort with CM and SM; mixed disease burden	KIT D816V testing; TPSAB1 genotyping	HαT associated with heightened symptom severity and diagnostic complexity	Supportive
Italian multicenter study [61]	Patients with MMAS or mastocytosis; enriched for BMM	Bone marrow-based diagnostics; TPSAB1 genotyping	BMM patients disproportionately represented among HαT+; more symptomatic and prone to anaphylaxis	Supportive
Korosec et al. [63]	Multicenter study across Central European HVA referral centers	Peripheral blood KIT D816V ASO-qPCR; TPSAB1 genotyping	HαT associated with lower bone marrow mast cell burden and fewer diagnostic features	Nuanced
González-de-Olano et al. [62]	Large Spanish REMA cohort (*n* = 959); SM, MMAS, and controls	Peripheral blood KIT D816V ASO-qPCR; TPSAB1 genotyping	HαT+ patients more likely to present with anaphylaxis, but similar recurrence rates; no increased HαT prevalence among anaphylaxis cases	Neutral
Chollet et al. [19]	A retrospective analysis conducted at a U.S. academic allergy center, as part of the PIONEER study	Standardized ISM criteria; TPSAB1 genotyping	No significant difference in anaphylaxis rates between HαT + and HαT– SM patients	Contradictory
Navarro-Navarro et al. [66]	Spanish Reference Center using ultra-sensitive KIT D816V detection	Ultra-sensitive KIT D816V detection in bone marrow and blood	Refined diagnostics increased detection of clonality in HαT+ patients; HαT less frequent in advanced SM	Diagnostic Confounder

HαT, hereditary alpha-tryptasemia; MMAS, monoclonal mast cell activation syndrome; BMM, bone marrow mastocytosis; SBT, SM, systemic mastocytosis; VIA, venom-induced anaphylaxis; HVA, Hymenoptera venom allergy

## Clinical Implications

HαT represents a genetic trait that can influence both the clinical presentation and diagnostic pathway in mast cell disorders, particularly in indolent forms such as ISM, BMM, and MMAS. Clinically, HαT has been associated with increased symptom burden in some cohorts—especially regarding cardiovascular manifestations and Hymenoptera venom allergy-related anaphylaxis—even though this relationship is not consistently observed across all populations. This variability underscores the importance of viewing HαT as a potential, but not definitive, clinical modifier.

From a diagnostic standpoint, HαT complicates the interpretation of elevated SBT levels, which is often used as a screening marker for clonal mast cell disease. Elevated SBT due to HαT may mimic mast cell activation or obscure the biochemical signature of clonality, potentially delaying diagnosis or leading to unnecessary procedures. Furthermore, tryptase dynamics during acute events may be harder to interpret in HαT-positive individuals, as genetically elevated baseline levels can mask pathological rises.

Moreover, the sensitivity of molecular techniques used to detect KIT D816V mutations plays a critical role in accurately identifying clonality, particularly in patients with low mast cell burden. False-negative results from peripheral blood testing may lead to underdiagnosis, especially in HαT-positive individuals where clinical suspicion remains high despite negative molecular findings.

Clinicians should consider HαT in patients presenting with unexplained mediator-related symptoms, particularly when tryptase levels are borderline and clonality testing via peripheral blood is negative. While HαT itself does not require treatment, its recognition can guide more targeted diagnostic strategies, support risk assessment, and help avoid misdiagnosis or over-investigation.

In addition to its potential association with mastocytosis, HαT has also been linked to MCAS, and a distinct HαT-MCAS variant has even been proposed. However, HαT can co-occur across all MCAS subtypes—including clonal, secondary, and idiopathic forms—making it conceptually inconsistent to define a separate HαT-MCAS entity. Rather than constituting a distinct variant, HαT should be considered a genetic trait whose influence on the clinical expression and severity of MCAS—regardless of its underlying classification—remains a matter of ongoing debate.

## Concluding Remarks

The evolving understanding of HαT in mastocytosis reveals a complex interplay between genetic predisposition and clonal mast cell pathology. While some studies suggest that HαT may amplify symptom severity in some patients, particularly those with low mast cell burden forms, its impact is not uniform and may vary depending on diagnostic sensitivity, referral patterns, and disease subtype.

Importantly, HαT challenges conventional diagnostic algorithms by confounding tryptase-based assessments and masking or mimicking features of mast cell activation. Genetic testing for TPSAB1 copy number variation should be considered in cases of elevated SBT with ambiguous clinical findings, especially when the likelihood of clonal disease is low.

Future population-based studies employing ultra-sensitive molecular techniques and stratified analyses are needed to elucidate the biological and clinical significance of HαT in mast cell disorders. Ultimately, HαT should be considered as one of several factors influencing the clinical expression of mastocytosis. Its presence warrants careful consideration, but should not be interpreted in isolation when making diagnostic or therapeutic decisions. Such efforts will be essential to disentangle the biological interplay between HαT and mast cell clonality, thereby refining diagnostic algorithms and informing therapeutic strategies.

## Key references

- Chovanec J, Tunc I, Hughes J, Halstead J, Mateja A, Liu Y, et al. Genetically defined individual reference ranges for tryptase limit unnecessary procedures and unmask myeloid neoplasms. Blood Adv. 2023;7(9):1796–810.
  - ○First cloning of the duplicated human tryptase locus and generated a model for what the upper limit of serum tryptase levels are based upon TPSAB1 replication number.
- Le QT, Lyons JJ, Naranjo AN, Olivera A, Lazarus RA, Metcalfe DD, et al. Impact of naturally forming human alpha/beta-tryptase heterotetramers in the pathogenesis of hereditary alpha-tryptasemia. J Exp Med. 2019;216(10):2348–61.
  - ○ First demonstration of naturally forming heterotetrameric tryptases and their unique activities in vitro. In addition, to date this remains the only study suggesting a potential functional role for α-tryptase, and further studies are needed to replicate these findings.
- Lyons JJ, Chovanec J, O’Connell MP, Liu Y, Šelb J, Zanotti R, et al. Heritable risk for severe anaphylaxis associated with increased α-tryptase-encoding germline copy number at TPSAB1 J Allergy Clin Immunol. 2021;147(2):622 − 32.
  - ○ First study to link HαT with mastocytosis, venom allergy, and idiopathic anaphylaxis.
- González-de-Olano D, Navarro-Navarro P, Muñoz-González JI, Sánchez-Muñoz L, Henriques A, de-Andrés-Martín A, et al. Clinical impact of the TPSAB1 genotype in mast cell diseases: A REMA study in a cohort of 959 individuals. Allergy. 2024 Mar;79(3):711–723. doi: 10.1111/all.15911.
  - ○ This is the first study to demonstrate that HαT does not consistently correlate with increased anaphylaxis risk, challenging its role as a universal clinical enhancer.
- Korosec P, Sturm GJ, Lyons JJ, Marolt TP, Svetina M, Kosnik M, et al. High burden of clonal mast cell disorders and hereditary alpha-tryptasemia in patients who need Hymenoptera venom immunotherapy. Allergy. 2024;79(9):2458-69. doi: 10.1111/all.16084.
  - ○ This study demonstrated that HαT may be associated with attenuated mast cell histopathology, challenging its presumed role as a consistent clinical amplifier.
- Navarro-Navarro P, González-Tablas M, Pérez-Pons A, Sánchez-Muñoz L, Henriques A, Álvarez-Twose I, et al. Improved diagnostic screening and classification of clonal mast cell diseases by ultrasensitive KIT p.D816V detection. Blood. 2025 Aug 14:blood.2025029507. doi: 10.1182/blood.2025029507. Epub ahead of print.
  - ○ The study demonstrated that ultrasensitive KIT D816V testing reveals stronger HαT–SM associations, especially in low-burden clonal mast cell diseases, reshaping diagnostic accuracy and interpretation.

## Data Availability

No datasets were generated or analysed during the current study.
